# Impact of the dosimetry approach on the resulting ^90^Y radioembolization planned absorbed doses based on ^99m^Tc-MAA SPECT-CT: is there agreement between dosimetry methods?

**DOI:** 10.1186/s40658-020-00343-6

**Published:** 2020-12-07

**Authors:** Verónica Morán, Elena Prieto, Lidia Sancho, Macarena Rodríguez-Fraile, Leticia Soria, Arantxa Zubiria, Josep M. Martí-Climent

**Affiliations:** 1 Department of Medical Physics and Radiation Safety, Clínica Universidad de Navarra, Madrid, Spain; 2 Department of Medical Physics and Radiation Safety, Clínica Universidad de Navarra, Pamplona, Spain; 3 IdisNA, Navarra Institute for Health Research, Pamplona, Spain; 4 Department of Nuclear Medicine, Clínica Universidad de Navarra, Madrid, Spain; 5 Department of Nuclear Medicine, Clínica Universidad de Navarra, Pamplona, Spain; 6 Department of Radiation Oncology, Clínica Universidad de Navarra, Pamplona, Spain

**Keywords:** ^90^Y-Microspheres, ^99m^Tc-MAA, Radioembolization, Predictive dosimetry, Partition model, Multi-tumor partition model, 3D voxel dosimetry, Local deposition method, Dose point kernel

## Abstract

**Background:**

Prior radioembolization, a simulation using ^99m^Tc-macroaggregated albumin as ^90^Y-microspheres surrogate is performed. Gamma scintigraphy images (planar, SPECT, or SPECT-CT) are acquired to evaluate intrahepatic ^90^Y-microspheres distribution and detect possible extrahepatic and lung shunting. These images may be used for pre-treatment dosimetry evaluation to calculate the ^90^Y activity that would get an optimal tumor response while sparing healthy tissues.

Several dosimetry methods are available, but there is still no consensus on the best methodology to calculate absorbed doses. The goal of this study was to retrospectively evaluate the impact of using different dosimetry approaches on the resulting ^90^Y-radioembolization pre-treatment absorbed dose evaluation based on ^99m^Tc-MAA images.

**Methods:**

Absorbed doses within volumes of interest resulting from partition model (PM) and 3D voxel dosimetry methods (3D-VDM) (dose-point kernel convolution and local deposition method) were evaluated. Additionally, a new “Multi-tumor Partition Model” (MTPM) was developed. The differences among dosimetry approaches were evaluated in terms of mean absorbed dose and dose volume histograms within the volumes of interest.

**Results:**

Differences in mean absorbed dose among dosimetry methods are higher in tumor volumes than in non-tumoral ones. The differences between MTPM and both 3D-VDM were substantially lower than those observed between PM and any 3D-VDM. A poor correlation and concordance were found between PM and the other studied dosimetry approaches.

DVH obtained from either 3D-VDM are pretty similar in both healthy liver and individual tumors. Although no relevant global differences, in terms of absorbed dose in Gy, between both 3D-VDM were found, important voxel-by-voxel differences have been observed.

**Conclusions:**

Significant differences among the studied dosimetry approaches for ^90^Y-radioembolization treatments exist. Differences do not yield a substantial impact in treatment planning for healthy tissue but they do for tumoral liver.

An individual segmentation and evaluation of the tumors is essential. In patients with multiple tumors, the application of PM is not optimal and the 3D-VDM or the new MTPM are suggested instead. If a 3D-VDM method is not available, MTPM is the best option. Furthermore, both 3D-VDM approaches may be indistinctly used.

**Supplementary Information:**

The online version contains supplementary material available at 10.1186/s40658-020-00343-6.

## Background

^90^Y-Radioembolization (RE) is an established treatment modality for patients with unresectable hepatic malignancies [1–4]. ^90^Y-loaded microspheres are injected selectively into the hepatic arteries that supply the tumors and permanently trapped in capillaries, delivering a high radiation absorbed dose to the tumor, while limiting the dose to non-tumoral liver parenchyma [5].

Prior treatment administration, a simulation is performed in order to assess ^90^Y-micospheres intrahepatic distribution and detect possible extrahepatic and lung shunting. For that purpose, ^99m^Tc-macroaggregated albumin (MAA) are selectively infused through the most appropriate hepatic arteries—selected after hepatic arteriography—to simulate intra- and extrahepatic ^90^Y-micospheres distribution. Within an hour after the ^99m^Tc-MAA administration, gamma scintigraphy is acquired and SPECT or SPECT-CT scans are recommended [6, 7] to allow a proper evaluation of intra and extrahepatic distributions, as well as to perform dosimetry evaluation to determine the most adequate ^90^Y activity that maximize tumor response while maintaining radiation exposure to surrounding healthy tissues within acceptable safety limits [8]. Thus, a treatment planning based on a proper dosimetry estimation has an important role in the optimization of the RE outcomes [9].

To date, several dosimetry approaches have been used for the calculation of ^90^Y-RE absorbed doses. Currently, for ^90^Y-resin microspheres (SIR-Spheres®; Sirtex Medical Limited, Australia), the most widely used method is PM [10], a multi-compartmental method based on the Medical internal Radiation Dose (MIRD) approach [11]. Mean absorbed doses (*D*_mean_) are calculated for 3 compartments (aggregated tumor, liver, and lung) assuming uniform distribution within each compartment. However, it must be taken into consideration that in most cases, microspheres distribution in tumoral and non-tumoral liver is not uniform. 3D-VDM methods, in contrast to PM, take into account inhomogeneities due to different intrahepatic distribution of the microspheres not only among individual tumors composing the aggregated tumor but also within each tumor.

In this work, a new multi-compartmental method called MTPM, which allows to calculate *D*_mean_ within each individual tumor, was developed and implemented. This dosimetry approach considers individual tumors as different compartments in order to take into account the heterogeneity among them.

The aim of this study was to retrospectively investigate and compare different dosimetry methods applied to ^90^Y RE pre-treatment planning, including the original MTPM approach. For that purpose, absorbed doses within volumes of interest (VOIs) resulting from multi-compartmental methods (PM and MTPM) were compared to those resulting from 3D-VDM methods (dose-point kernel (DPK) convolution and local deposition method (LDM)). The differences among the dosimetry approaches were evaluated within different VOIs in terms of *D*_mean_ and dose volume histograms (DVH).

## Methods

### Patient characteristics

Fourteen patients with hepatic malignancies who underwent RE with ^90^Y-resin microspheres in our institution from 2013 to 2015 were retrospectively evaluated. The inclusion criteria were availability of a contrast-enhanced CT or MRI within 4 weeks prior to treatment, lesions that could be unequivocally delineated, and similar positioning of the catheter both in the simulation with ^99m^Tc-MAA and in the therapeutic ^90^Y-microespheres administration. No other clinical or demographic data was taken into account for the patient selection because it is not required to achieve the principal aim of this study: to compare dosimetry methods.

### (^99m^Tc-MAA) protocol scan and activity planning

Once ^99m^Tc-MAA were injected trough the selected arteries during hepatic arteriography, planar and SPECT-CT images were acquired in a Symbia T2 (Siemens Medical Solutions, Erlangen, Germany) with a dual-head variable-angle gammacamera and a two-slice spiral CT scanner. A low-energy high-resolution (LEHR) collimator was used with an energy window centered at 140 keV and 15% wide.

For planar imaging, anterior and posterior images of the abdomen and the thorax (10-min acquisition) were taken in a 128 × 128 matrix. No zoom was applied.

For SPECT acquisition, 128 images (20 s per projection) were acquired over 360° using a 128 × 128 matrix with a pixel size of 4.8 × 4.8 mm^2^. Images were reconstructed using a Flash 3D algorithm (8 iterations, 4 subsets, 8.4 mm FWHM Gaussian post-filter), an iterative algorithm considering a 3D collimator beam modeling, CT-based attenuation correction, and energy window-based scatter correction. The scan parameters for CT were 130 kV, 25 mAs, and 5-mm slices. Both SPECT and CT images were fused using an Esoft 2000 application package (Siemens Medical Solution, Erlangen, Germany).

As previously published by Gil-Alzugaray et al. [12], in our center, the administered ^90^Y activity was planned by means of PM for lobar and segmental treatments and by body surface area model for whole liver treatments. This methods were applied according to the microspheres’ manufacturer recommended guidelines [13].

The lung shunt fraction (LSF) was calculated by Eq. (1), where *C*_lung_ and *C*_WL_ are the geometric mean of total counts (anterior and posterior images) registered within lungs and whole liver, respectively:

1 $$ \mathrm{LSF}\left(\%\right)=100\bullet \frac{C_{\mathrm{lung}}}{C_{\mathrm{lung}}+{C}_{\mathrm{WL}}} $$

Planar images may not be used to determine accurately the tumor to non-tumor liver activity concentration ratio (TNR) [14]; therefore, attenuation-corrected SPECT images were used instead. TNR was calculated by Eq. (2), where *C*_TL_ and *C*_NLt_ are the total counts registered within TL and NL_*t*_ volumes respectively:

2 $$ \mathrm{TNR}=\frac{C_{\mathrm{TL}}/{V}_{\mathrm{TL}}}{{\mathrm{C}}_{\mathrm{N}{\mathrm{L}}_{\mathrm{t}}}/{\mathrm{V}}_{\mathrm{N}{\mathrm{L}}_{\mathrm{t}}}} $$

### Contouring

The first step for this retrospective investigation was the anatomic VOIs segmentation. These VOIs were contoured on the CT from ^99m^Tc-MAA SPECT-CT with the aid of a rigidly registered diagnostic scan (contrast enhanced CT or MRI) using a commercial treatment planning software (Pinnacle, Philips Medical System, Anover, MA). A process similar to the one used in external beam radiation therapy was followed. The VOIs were then exported as DICOM-RT structure sets. To avoid inter-operator bias, all VOIs were delineated by a single physician.

For each patient, individual tumors (T_i_), the planning target volume (PTV), and the whole liver (WL) were delineated. The PTV refers to the portion of the liver in which it is intended to deliver the radiation dose: one or more segments, one lobe or the whole liver depending on whether the treatment is segmental, lobar, or total. Tumoral liver volume (TL), corresponding to the aggregated tumor volume was generated by summing all the T_i_ volumes. Target normal liver volume (NL_t_) was defined by subtracting the TL volume from the PTV volume. Whole normal liver (NL_w_) was also determined by subtracting the TL volume from the WL. Volumes in mL for individual tumors, aggregated tumoral liver, target normal liver, and whole normal liver (V_Ti_, V_TL_, ${V}_{\mathrm{N}{\mathrm{L}}_{\mathrm{t}}}$, and ${V}_{\mathrm{N}{\mathrm{L}}_{\mathrm{w}}}$) were calculated for the 14 patients.

### Dosimetry assessment

For the purposes of this study, the mean absorbed dose delivered to each compartment (${D}_{\mathrm{mean}}^{{\mathrm{T}}_{\mathrm{i}}},{D}_{\mathrm{mean}}^{\mathrm{T}\mathrm{L}},{D}_{\mathrm{mean}}^{\mathrm{N}{\mathrm{L}}_{\mathrm{t}}}$, and${\mathrm{D}}_{\mathrm{mean}}^{\mathrm{N}{\mathrm{L}}_{\mathrm{w}}}$) was estimated according to MIRD formalism [11]. Both multi-compartment dosimetry methods were retrospectively applied to obtain *D*_Mean_. To implement MTPM, in patients with two individual tumors or more, an Excel-based mean absorbed dose calculator was developed (available in additional file 1).

Additionally, DPK and LDM were applied to calculate a 3D dose map and DVHs. The actual ^90^Y administered activity and volumes of the contoured VOIs used to determine the absorbed doses were the same for all dosimetry approaches (PM, MTPM, and both 3D-VDM).

For the ^90^Y dosimetry calculation purposes, an identical ^99m^Tc-MAA and ^90^Y-microspheres’ biodistributions were assumed, based on previous studies [10, 15–19].

#### Multicompatimental methods

PM was applied to calculate ${D}_{\mathrm{Mean}}^{\mathrm{TL}}$ and ${D}_{\mathrm{Mean}}^{\mathrm{N}{\mathrm{L}}_{\mathrm{t}}}$ according to Eqs. (3) and (4), where A(^90^Y) is the ^90^Y-microspheres administered activity, and M_TL_ and ${M}_{N{L}_t}$are the masses in kg of the tumoral liver and the target normal liver, respectively. A 1 g/mL tissue density is assumed, and volumes in liters are straight converted in masses in kg.

${D}_{\mathrm{Mean}}^{\mathrm{N}{\mathrm{L}}_{\mathrm{w}}}$ was determined by rescaling the ${D}_{\mathrm{Mean}}^{\mathrm{N}{\mathrm{L}}_{\mathrm{t}}}$ to the NL_w_volume, applying (5).

3 $$ {D}_{\mathrm{Mean}}^{\mathrm{TL}}(Gy)=\frac{49.67\ \left(\frac{J}{GBq}\right)\bullet A\Big({}^{90}Y\Big)(GBq)\left(1-\raisebox{1ex}{$ LSF$}\!\left/ \!\raisebox{-1ex}{$100$}\right.\right)\bullet \mathrm{TNR}}{M_{\mathrm{TL}}(kg)\bullet \mathrm{TNR}+{M}_{\mathrm{N}{\mathrm{L}}_{\mathrm{t}}}(kg)} $$

4 $$ {D}_{\mathrm{Mean}}^{\mathrm{N}{\mathrm{L}}_{\mathrm{t}}}(Gy)=\frac{49.67\left(\frac{J}{GBq}\right)\bullet A\Big({}^{90}Y\Big)(GBq)\left(1-\raisebox{1ex}{$ LSF$}\!\left/ \!\raisebox{-1ex}{$100$}\right.\right)}{M_{\mathrm{TL}}(kg)\bullet \mathrm{TNR}+{M}_{\mathrm{N}{\mathrm{L}}_{\mathrm{t}}}(kg)} $$

5 $$ {D}_{\mathrm{Mean}}^{\mathrm{N}{\mathrm{L}}_{\mathrm{w}}}(Gy)=\frac{D_{\mathrm{N}{\mathrm{L}}_{\mathrm{t}}}(Gy)\bullet {M}_{\mathrm{N}{\mathrm{L}}_{\mathrm{t}}}(kg)}{M_{\mathrm{N}{\mathrm{L}}_{\mathrm{w}}}(kg)} $$

In patients with two or more tumors (*n*), the MTPM method, an (*n* + 2) compartment partition model, was applied to determine ${D}_{\mathrm{Mean}}^{{\mathrm{T}}_{\mathrm{i}}}$using Eq. (6), where TNR_i_ is the tumor to normal liver activity concentration ratio for individual tumors calculated by Eq. (7).

6 $$ {D}_{\mathrm{M}\mathrm{ean}}^{{\mathrm{T}}_{\mathrm{i}}}(Gy)=\frac{49.67\ \left(\frac{J}{GBq}\right)\bullet A\Big({}^{90}Y\Big)(GBq)\left(1-\raisebox{1ex}{$ LSF$}\!\left/ \!\raisebox{-1ex}{$100$}\right.\right)\bullet TN{R}_i}{{\mathrm{M}}_{\mathrm{T}\mathrm{L}}(Kg)\bullet \mathrm{TNR}+{M}_{\mathrm{N}{\mathrm{L}}_{\mathrm{t}}}(Kg)} $$

7 $$ \mathrm{TN}{\mathrm{R}}_i=\frac{C_{{\mathrm{T}}_{\mathrm{i}}}/{V}_{{\mathrm{T}}_{\mathrm{i}}}}{C_{\mathrm{N}{\mathrm{L}}_{\mathrm{t}}}/{V}_{\mathrm{N}{\mathrm{L}}_{\mathrm{t}}}} $$

#### 3D-voxel dosimetry

The first step to perform 3D image-based dosimetry using ^99m^Tc-MAA SPECT is to convert, through a calibration factor, the counts registered in each voxel of the reconstructed image to ^90^Y activity (in MBq). Since ^99m^Tc-MAA administered activity (A(^99m^Tc)) is totally uptaken in the liver with the exception of the fraction that shunts to the lung, the patient-specific calibration factor may be determined as it was described by Chiesa et al. [20].

The ^90^Y-microspheres activity in a liver voxel at the image acquisition time (A_voxel_(^90^Y)) is directly proportional to the total counts registered within a voxel of ^99m^Tc-MAA SPECT image (*C*_voxel_(^99m^Tc)). Thus, A_voxel_(^90^Y) may be estimated by means of Eq. (8) where *C*_WL_ is the total counts of ^99m^Tc registered within the WL volume.

8 $$ {A}_{\mathrm{voxel}}\left({}^{90}Y\right)={C}_{\mathrm{voxel}}\left({}^{99m} Tc\Big)\frac{A\ \left({}^{99m} Tc\right)\left(1-\frac{LSF}{100}\right)}{C_{WL}\left({}^{99m} Tc\right)}\frac{A\Big({}^{90}Y\Big)}{A\Big({}^{99m} Tc\Big)}={C}_{\mathrm{voxel}}\right({}^{99m} Tc\Big)\frac{\left(1- LSF/100\right)A\Big({}^{90}Y\Big)}{C_{WL}\left({}^{99m} Tc\right)} $$

Unlike other internal radionuclide therapy, RE has the advantage of negligible biological clearance following the infusion. Thus, assuming the permanent trapping of microspheres, fitting of time-activity curves is not required, and the total number of disintegrations in a voxel (Ã _voxel_(^9o^Y)) was calculated as described by Eq. (9), where *T*_1/2_(^90^*Y*) is the physical ^90^Y half-life (64.2 h).

9 $$ {\overset{\sim }{A}}_{\mathrm{voxel}}\left({}^{90}Y\right)=\int {A}_{\mathrm{voxel}}\left({}^{90}Y\right)\bullet {e}^{\left(\raisebox{1ex}{$- Ln(2)t$}\!\left/ \!\raisebox{-1ex}{${T}_{\frac{1}{2}}$}\right.\right)}\bullet dt=1.443\bullet {T}_{\raisebox{1ex}{$1$}\!\left/ \!\raisebox{-1ex}{$2$}\right.}\left({}^{90}Y\right).\kern0.5em {A}_{\mathrm{voxel}}\left({}^{90}Y\right) $$

To convert the cumulative activity in each voxel to a tridimensional ^90^Y absorbed dose map, two different 3D-VDM approaches were applied: LDM and DPK. For that purpose, a software tool based in MATLAB v.R2016a (The Math Works, Natick, MA) code was developed.

DPK takes into account the high-energy beta particles transport to adjacent voxels. The absorbed dose within the target voxel t $\left({D}_{\mathrm{voxe}{\mathrm{l}}_{\mathrm{t}}}\left({}^{90}Y\right)\right)$ was calculated by the convolution of the 3D cumulative activity matrix with a cubic dose kernel, as described in Eq. (10). Where ${\overset{\sim }{A}}_{\mathrm{voxe}{\mathrm{l}}_{\mathrm{s}}}\left({}^{90}Y\right)$ is the time-integrated activity within the source voxel *s,* and *S*(voxel_t_ ← voxel_s_) is the well-known *S* value defined as the absorbed dose to the target voxel *t* per unit of cumulative activity in the voxel *s*. The dose kernels used in this work were extracted from Lanconelli database [21].

10 $$ {D}_{\mathrm{voxe}{\mathrm{l}}_{\mathrm{t}}}\left({}^{90}Y\right)=\sum \limits_{s=0}^N{\overset{\sim }{A}}_{\mathrm{voxe}{\mathrm{l}}_{\mathrm{s}}}\left({}^{90}Y\right)\otimes S\left(\mathrm{voxe}{\mathrm{l}}_{\mathrm{t}}\leftarrow \mathrm{voxe}{\mathrm{l}}_{\mathrm{s}}\right) $$

LDM assumes that the kinetic energy from each beta particle is deposited within the voxel where the emission occurs. The source voxel *s* in this case is also the target voxel *t*. The absorbed dose in each voxel was then determined by Eq. (11), multiplying the cumulative activity within the voxel by a constant scalar factor, which is the *S* value considering an absorbed fraction equal to 1 in each voxel (${\left.S\left(\mathrm{voxe}{\mathrm{l}}_{\mathrm{t}}\leftarrow \mathrm{voxe}{\mathrm{l}}_{\mathrm{s}}\right)\right|}_{\mathrm{voxe}{\mathrm{l}}_{\mathrm{t}}=\mathrm{voxe}{\mathrm{l}}_{\mathrm{s}}}\Big)$. *S* is calculated by means of Eq. (12), where ${\left\langle {E}_{\beta}\left({}^{90}Y\right)\right\rangle}_{\mathrm{voxe}{\mathrm{l}}_{\mathrm{t}}}=\left(\frac{0.9267\  MeV}{\mathrm{disintegration}}\right)\bullet \left(\frac{1.6022\bullet {10}^{-13}J}{MeV}\right)\bullet \left(\frac{Gy\bullet Kg}{J}\right)\bullet \left(\frac{10^9\mathrm{disintegrations}}{s\bullet GBq}\right)$ is the deposited β-energy per disintegration in average, and ${M}_{\mathrm{voxe}{\mathrm{l}}_{\mathrm{t}}}$is the target voxel mass. For a given cubic voxel size (4.48 mm side), *S* is 1.603 Gy/GBq.s.

11 $$ {\left.{D}_{\mathrm{voxe}{\mathrm{l}}_{\mathrm{t}}}\left({}^{90}Y\right)={\overset{\sim }{A}}_{\mathrm{voxe}{\mathrm{l}}_{\mathrm{s}}}\left({}^{90}Y\right)\times S\left(\mathrm{voxe}{\mathrm{l}}_{\mathrm{t}}\leftarrow \mathrm{voxe}{\mathrm{l}}_{\mathrm{s}}\right)\right|}_{\mathrm{voxe}{\mathrm{l}}_{\mathrm{t}}=\mathrm{voxe}{\mathrm{l}}_{\mathrm{s}}} $$

12 $$ {\left.S\left(\mathrm{voxe}{\mathrm{l}}_{\mathrm{t}}\leftarrow \mathrm{voxe}{\mathrm{l}}_{\mathrm{s}}\right)\right|}_{\mathrm{voxe}{\mathrm{l}}_{\mathrm{t}}=\mathrm{voxe}{\mathrm{l}}_{\mathrm{s}}}={\left.\frac{{\left\langle {\mathrm{E}}_{\upbeta}\left({}^{90}\mathrm{Y}\right)\right\rangle}_{\mathrm{voxe}{\mathrm{l}}_{\mathrm{t}}}}{M_{\mathrm{voxe}{\mathrm{l}}_{\mathrm{t}}}}\ \right|}_{\mathrm{voxe}{\mathrm{l}}_{\mathrm{t}}=\mathrm{voxe}{\mathrm{l}}_{\mathrm{s}}} $$

### Dosimetry comparisons and statistical analysis

${D}_{\mathrm{mean}}^{\mathrm{N}{\mathrm{L}}_{\mathrm{t}}}$ and ${D}_{\mathrm{mean}}^{\mathrm{N}{\mathrm{L}}_{\mathrm{w}}}$calculated by PM, LDM, and DPK methods were compared using a paired Student’s *t* test or Wilcoxon test in case differences between methods do not meet normal criteria.

${D}_{\mathrm{mean}}^{{\mathrm{T}}_{\mathrm{i}}}$ and ${D}_{\mathrm{mean}}^{\mathrm{TL}}$calculated by PM, MTPM, and both 3D-VDM were also compared using a paired Student’s *t* test or Wilcoxon test, as corresponds. For MTPM, LDM, and DPK, ${D}_{\mathrm{mean}}^{\mathrm{TL}}$ was calculated for each patient as the average of all ${D}_{\mathrm{mean}}^{\mathrm{Ti}}$. The standard deviation (SD) was also determined. ${D}_{\mathrm{mean}}^{\mathrm{Ti}}$ calculated by PM was the same for all individual tumors of the same patient, and equal to ${D}_{\mathrm{mean}}^{\mathrm{TL}}$, as tumoral liver compartment in PM approach is defined as an aggregated tumor including all T_i_.

The heterogeneity of ^90^Y-microspheres distribution among the tumors for each patient was evaluated through the TNR_i_ coefficient of variation (COV(TNR_i_)).

A comparison among the studied dosimetry methods for all VOIs was performed in terms of mean absorbed dose differences ($\Delta  {D}_{\mathrm{mean}}^{\mathrm{VOI}}$) in Gy.

The correlation between differences in *D*_mean_ between PM and the other studied dosimetry methods (MTPM, LDM, and DPK) and TNR-TNR_i_ differences was evaluated by means of the Spearman’s correlation coefficient (rho).

Dosimetry comparison between DPK and LDM methods was also managed in terms of DVHs. Some metrics were extracted from the DVHs: the minimum dose to 5%, 25%, 50%, 70%, and 95% in the corresponding VOI (*D*_5_, *D*_25_, *D*_50_, *D*_70_, and *D*_95_, respectively), the percentage of the tumor volume receiving at least 100 Gy (V_100_) and the percentage of the NL_w_ and NL_t_ volumes receiving at least 20 Gy (V_20_). A paired Student’s *t* test or Wilcoxon test, as appropriate, was applied. Absorbed dose differences in Gy were also determined for each VOI.

Bland-Altman analysis was used to evaluate the agreement among the studied dosimetry methods (PM, MTPM, LDM, and DPK), in terms of *D*_mean_, for both tumoral and non-tumoral volumes (NL_t_, NL_w_, TL, and T_i_). The agreement of DVH between both 3D-VDM methods was also evaluated by means of a Bland-Altman analysis. Pearson correlation (*ρ*) and Lin concordance (ρ_c_) coefficients were reported.

All analyses were performed with statistical STATA v.15 software (StataCorp, TX, USA). A *p* value of 0.05 or less was considered statistically significant.

Differences between LDM and DPK methods were also assessed by a voxel by voxel analysis. A voxel based subtraction of the parametric images (in Gy) calculated by both methods was performed, and the calculation of the normalized mean square error (NMSE) between dose absorbed maps obtained applying Eq. (13), as described previously by Pacilio et al. [22], where *x*_*i*_ is the *i*th voxel of the DPK image and *p*_*i*_ the *i*th voxel of the LDM image (used as a reference).

13 $$ NMSE=100\bullet \frac{\sum \limits_i{\left({x}_i-{p}_i\right)}^2}{\sum \limits_i{p}_i^2} $$

## Results

Finally, 14 patients were collected according the inclusion criteria. There were 5 patients who received whole liver treatment, 8 who received lobar treatments (7 right and 1 left lobe) and 1 superselective treatment to one hepatic segment. In total, 101 individual tumors were identified and analyzed, with a volume range from 0.6 to 351 mL. Prior to RE, in order to simulate the ^90^Y-labeled microspheres biodistribution, 161 ± 11 MBq of ^99m^Tc-MAA were administered.

The treatment characteristics and volumes of contoured VOIs are reported in Table 1 for the 14 patients.

**Table 1:** Treatment characteristics: treatment approach, number of treated tumors, ^90^Y administered activity, the percentage of lung shunt fraction, tumor to non-tumor liver activity concentration ratio, and volumes for individual tumors (average ± SD), aggregated tumoral liver, target normal liver, and whole normal liver

Patient	Treatment approach	Number of tumors	^90^Y Activity (GBq)	LSF (%)	TNR	${V}_{T_i}$ (mL)	*V*_*TL*_ (mL)	${V}_{N{L}_t}$ (mL)	*V*_*NLw*_ (mL)
1	Whole-liver	4	1.4	6.1	1.0	143 ± 155	573	1875	1785
2	Lobar	2	1.3	1.4	0.6	2 ± 1	4	789	1235
3	Lobar	1	1.2	10.1	0.8	12	12	969	1509
4	Whole-liver	50	1.8	4.0	1.2	15 ± 33	735	2277	2277
5	Lobar	3	0.6	10.7	1.2	6 ± 1	18	410	1434
6	Lobar	1	0.5	6.6	0.6	93	93	932	1551
7	Whole-liver	9	0.9	1.8	1.7	6 ± 6	50	1176	1176
8	Lobar	1	1.1	9.0	2.3	282	282	982	1577
9	Lobar	2	1	3.9	2.3	13 ± 6	26	753	1240
10	Lobar	4	0.9	2.2	1.5	10 ± 5	42	887	1725
11	Whole-liver	15	1.6	5.3	2.2	12 ± 17	177	1605	1605
12	Segmental	2	1	4.1	1.4	14 ± 1	29	472	1205
13	Lobar	1	1.8	3.6	1.2	130	129	1003	1561
14	Whole-liver	6	1.3	5.8	0.7	82 ± 53	493	1373	1373

**Fig. 1:**
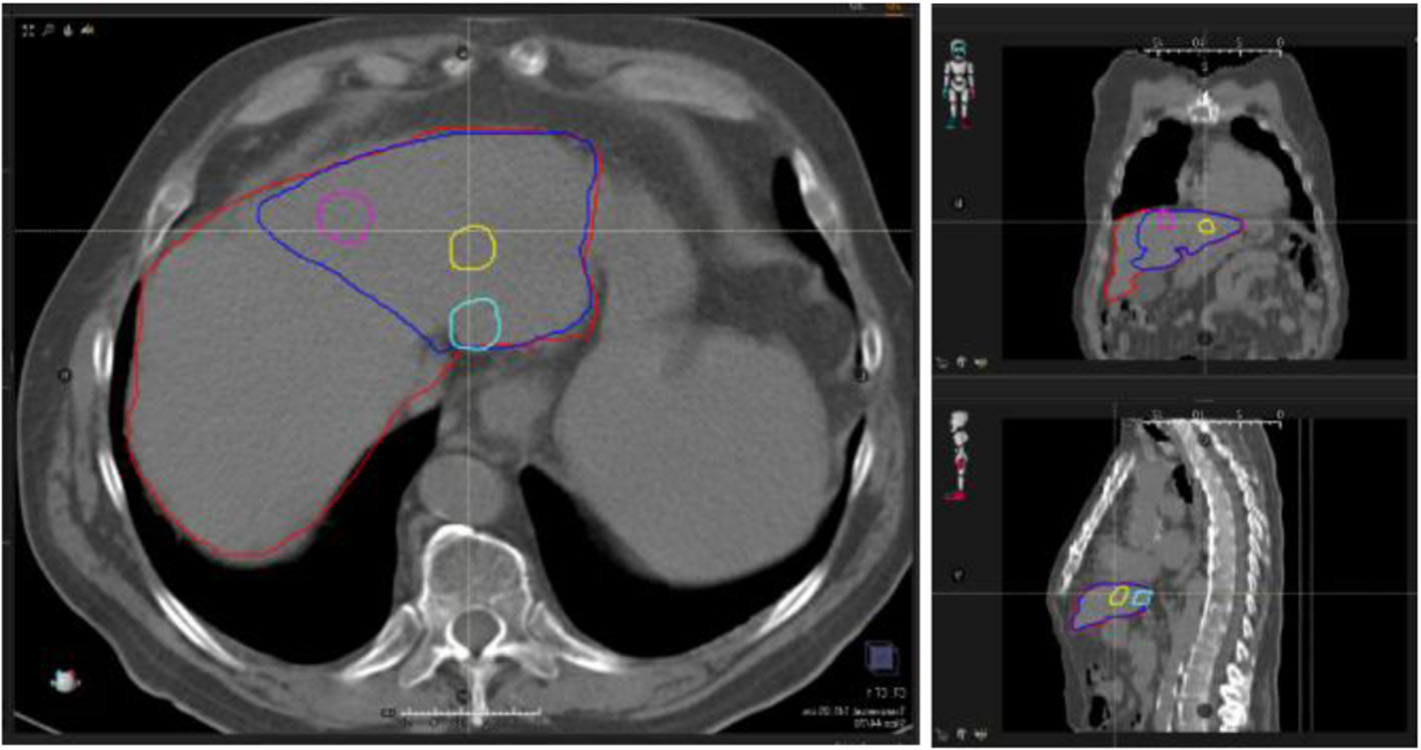
Volumes of interest contoured on the ^99m^Tc-MAA SPECT-CT images used for the pre-therapy dosimetry assessment corresponding to patient 12: whole liver volume delimited by red line, target volume delimited by blue line and individual tumors delimited by pink, yellow, and light blue lines

An example of ^99m^Tc-MAA SPECT-CT images used to perform the dosimetry calculation is reported in Fig. 1, as well as the delineated VOIs in axial, coronal, and sagittal planes.

### Absorbed dose by normal liver

**Fig. 2:**
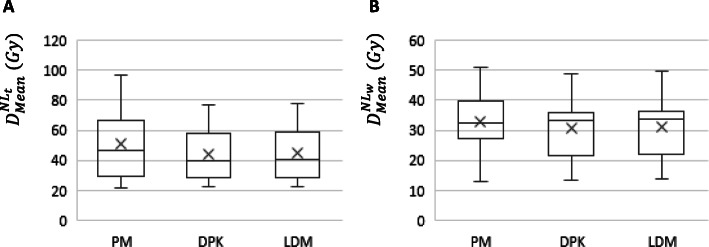
Mean absorbed doses delivered to target normal liver **a** and whole normal liver **b** volumes, determined by means of PM and both 3D voxel dosimetry methods (LDM and DPK)

Box plots summarizing ${D}_{\mathrm{mean}}^{\mathrm{N}{\mathrm{L}}_{\mathrm{t}}}$ and ${D}_{\mathrm{mean}}^{\mathrm{N}{\mathrm{L}}_{\mathrm{w}}}$, calculated by means of PM, DPK, and LDM methods, are shown in Fig. 2.

**Fig. 3:**
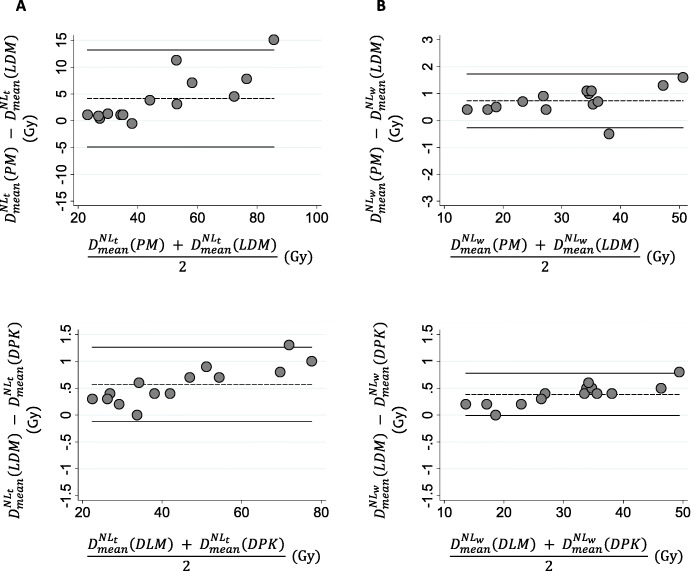
Bland-Altman diagrams representing the relative dose difference in Gy between dosimetry methods versus the mean absorbed dose values in Gy for both normal target liver **a** and whole normal liver **b**. The dashed line represents the average of the differences between the studied dosimetry methods, and the black line is the mean differences of ± 2 SD

To assess the agreement between the PM and LDM method and also between both 3D-VDM studied dosimetry methods, in terms of D_mean_, Bland-Altman plots are shown in Fig. 3 for both NL_t_ and NL_w_. The comparison between PM and DPK method are shown in additional file 2. Note the different scale according the compared methods. Pearson’s correlation and Lin concordance coefficients for each comparison between dosimetry methods are summarized in Table 2. For both $$ {D}_{mean}^{N{L}_t} $$ and $$ {D}_{mean}^{N{L}_w}, $$ 1/14 (7.1%) of the points are beyond the ±2 SD lines in all pair of comparisons. The PM method significantly overestimates *D*_mean_ with respect to LDM and DPK methods (*p* < 0.01). The *D*_mean_ determined by LDM is also higher than those calculated applying DPK. Maximum $$ {\Delta  D}_{mean}^{N{L}_w} $$ and $$ {\Delta  D}_{mean}^{N{L}_t} $$ between PM and 3D methods were 2.4 Gy and 16.1 Gy respectively, and between both 3D-VDM methods were 0.8 Gy for NL_w_ and 1.3 Gy for NL_t_. Significant differences were observed in $$ {D}_{mean}^{N{L}_t} $$ and $$ {D}_{mean}^{N{L}_w} $$between PM and 3D-VDM methods and also between both 3D-VDM methods (*p* value <0.01).

**Table 2:** Comparison among PM and both 3D dosimetry methods (LDM and DPK) in terms of mean absorbed dose delivered to target and whole normal liver: Bland Altman analysis, Pearson’s correlation, and Lin concordance coefficients

	Bland-Altman		Correlation and concordance coefficients	
	$\Delta {D}_{Mean}^{VOI}$ (Gy)	Bias [95%CI] (Gy)	*ρ*	ρ_c_
*Target normal liver*				
PM-DPK	4.7	− 4.8; 14.2	0.99	0.94
PM-LDM	4.2	− 4.9; 13.2	0.99	0.95
LDM-DPK	0.6	− 0.1; 1.3	1.00	1.00
*Whole normal liver*				
PM-DPK	1.1	− 0.1; 2.4	1.00	0.99
PM-LDM	0.7	− 0.3; 1.7	1.00	1.00
LDM-DPK	0.4	0.0; 0.8	1.00	1.00

**Fig. 4:**
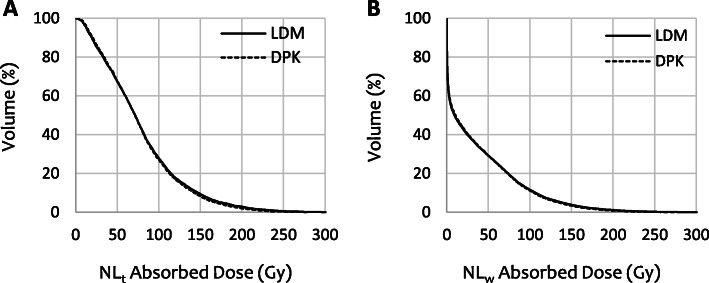
Dose volume histogram curves for both target normal liver **a** and whole normal liver volumes **b** corresponding to patient 12. Continuous lines correspond to LDM method and the dashed lines to DPK method

DVH curves calculated by means of LDM and DPK dosimetry methods for both NL_t_ and NL_w_ volumes are reported in Fig. 4. DVHs correspond to patient 12, who yielded one of the highest differences between both 3D-VDM methods.

**Fig. 5:**
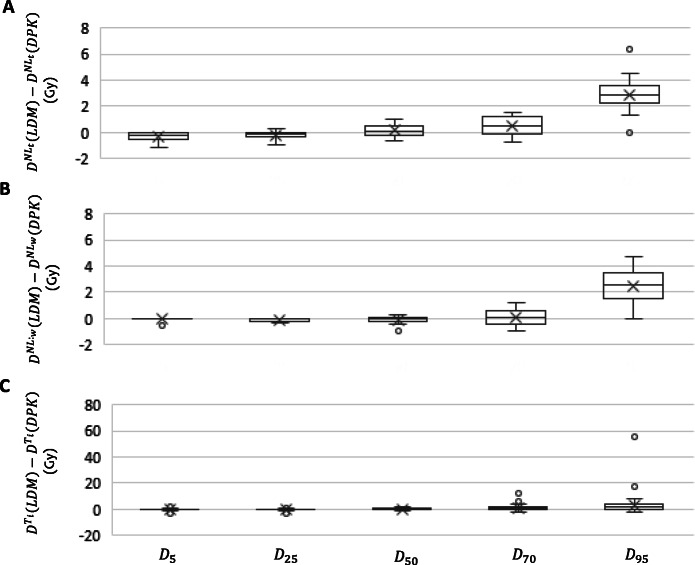
Boxplot representing absorbed dose differences in Gy between LDM and DPK methods in terms of D_5_, D_25_, D_50_, D_70_, and D_95_ delivered to the target normal liver **a**, whole normal liver **b**, and individual tumors **c**

The differences between LDM and DPK methods in DVH in terms of D_5_, D_25_, D_50_, D_70_, and D_95_ for the healthy liver volumes (NL_t_ and NL_w_) are reported in Fig. 5. Higher differences were found for D_70_ and D_95_ while D_50_, D_25_, and D_5_ showed lower variations in both compartments. The maximum differences for NL_t_ and NL_w_ were found for D_95_, with values of 6.34 and 4.71 Gy, respectively.

The statistical analysis showed that DVH differences between both 3D-VDM were statistically significant except for the following cases: D_25_ and D_50_ for NL_t_ (*p* value 0.06 and o.17) and D_5_, D_50_, and D_70_ for NL_w_ (*p* values: 0.26, 0.95, and 0.68).

The mean (range) difference in V_20 Gy_ (evaluated as %) due to applied LDM or DPK methods for both NL_t_ and NL_w_ were − 0.3 (− 1.0, 0.3) % and − 0.2 (− 0.6, 0.3) %, respectively. Significant differences were observed in both compartments (*p* value < 0.01).

The results of the Bland-Altman analysis to assess differences in DVH in terms of D_5_, D_25_, D_50_, D_70_, and D_95_ are summarized in Table 3, for both NL_t_ and NL_w_. Pearson’s correlation and Lin concordance coefficients are greater than 0.995 for all the comparisons. For both compartments, at most, 1/14 (7.1%) of the points are beyond the ± 2 SD lines for all the studied endpoints.

**Table 3:** Comparison among both 3D dosimetry methods (LDM and DPK) in terms of *D*_*5*_*, D*_*25*_*, D*_*50*_*, D*_*70*_*, and D*_*95*_ delivered to target normal liver, whole normal liver, and individual tumors: Bland Altman analysis and Lin concordance coefficients

	Bland-Altman			Correlation and concordance coefficients	
	*∆D* (Gy)	Bias [95%CI] (Gy)	Points beyond ± 2 SD (%)	*ρ*	ρ_c_
	Target normal liver				
D_5_	0.3	− 0.9; 0.4	7.1	0.999	0.999
D_25_	− 0.2	− 0.8; 0.4	7.1	1.000	1.000
D_50_	0.2	− 0.8; 1.1	0.0	1.000	1.000
D_70_	0.4	− 1.0; 1.9	0.0	1.000	0.999
D_95_	2.3	0.0; 5.9	7.1	1.000	0.996
	Whole normal liver				
D_5_	0.1	− 0.4; 0.3	7.1	0.999	0.998
D_25_	− 0.1	− 0.5; 0.2	7.1	1.000	1.000
D_50_	− 0.1	− 0.9; 0.7	7.1	1.000	1.000
D_70_	0.1	− 1.2; 1.3	0.0	1.000	1.000
D_95_	2.5	0.1; 4.9	7.1	1.000	0.996
	Individual tumors				
D_5_	− 0.3	− 1.6; 1.0	5.0	0.999	0.999
D_25_	− 0.1	− 1.7; 1.8	4.0	0.999	0.999
D_50_	0.4	− 1.7; 2.5	6.0	1.000	0.999
D_70_	1.1	− 2.3; 4.5	4.0	1.000	0.999
D_95_	3.1	− 9.0; 15.2	2.0	0.999	0.995

### Absorbed dose by tumors

**Fig. 6:**
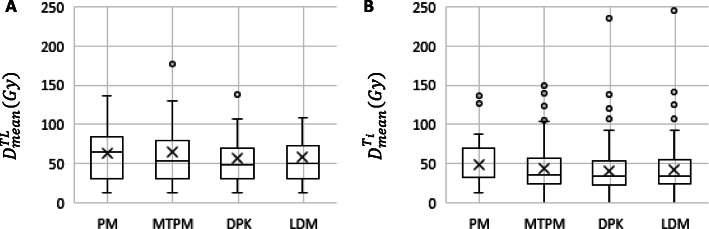
Mean absorbed doses delivered to the aggregated tumoral liver **a** and each individual tumor **b** volumes, determined by means of multicompartmental (PM and MTPM) and 3D voxel dosimetry methods (LDM and DPK)

Box plots summarizing ${D}_{mean}^{T_i}$ and ${D}_{mean}^{TL}$ calculated applying PM, MTPM, DPK, and LDM methods are shown in Fig. 6.

For patients with more than one single tumor, ${D}_{mean}^{TL}$ determined applying PM is summarized in Table 4, as well as the COV(TNR_i_). For MTPM, and both 3D-VDM ${D}_{mean}^{TL}$ is also showed as average of ${D}_{mean}^{T_i}$ ± SD. The maximum ${\Delta  D}_{mean}^{TL}$ among the studied methods for PM-MTPM, PM-DPK, PM-LDM, MTPM-DPK, MTPM-LDM, and LDM-DPK were 41.3, 41.5, 40.7, 39.7, 34.1, and 5.6 Gy, respectively. Significant differences were observed among the all dosimetry methods in terms of ${D}_{mean}^{TL}$ except for PM-MTPM (*p* value = 0.32).

**Table 4:** Mean absorbed dose delivered to aggregated tumoral liver calculated by means of multicompartmental (PM and MTPM) and 3D voxel dosimetry methods (LDM and DPK), for patients with more than a single lesion. COV(TNR_i_) is also reported

Patient	${D}_{mean}^{TL}(Gy)$				COV(TNRi)
	PM	MTPM	DPK	LDM	
1	28	26 ± 28	25 ± 27	25 ± 28	109
2	47	50 ± 11	44 ± 9	45 ± 10	22
4	32	33 ± 14	31 ± 13	32 ± 14	44
5	74	76 ± 36	66 ± 31	67 ± 32	47
7	60	56 ± 19	53 ± 17	54 ± 18	33
9	137	177 ± 178	137 ± 138	143 ± 144	100
10	70	75 ± 22	66 ± 20	69 ± 20	29
11	82	41 ± 47	41 ± 46	42 ± 47	113
12	127	129 ± 29	107 ± 26	108 ± 25	23
14	24	29 ± 10	28 ± 10	28 ± 10	36

**Fig. 7:**
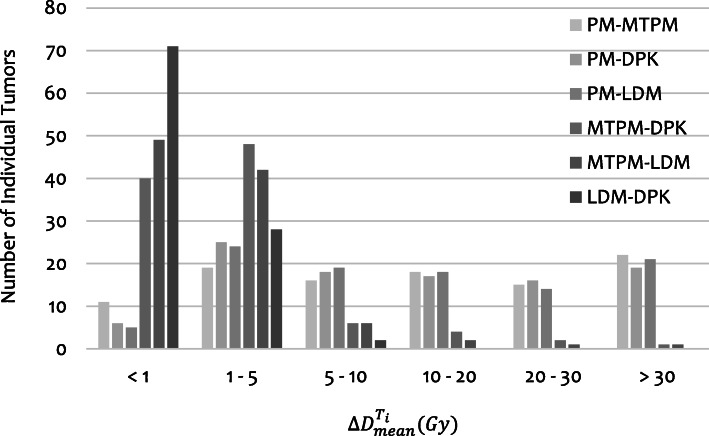
Distribution of differences in mean dose absorbed within individual tumors for the studied methods compared by pairs

In Fig. 7, the distribution of the ${\Delta \mathrm{D}}_{\mathrm{mean}}^{{\mathrm{T}}_{\mathrm{i}}}$for all dosimetry methods comparisons overall 101 individual tumors is shown. Differences between PM and the other methods (MTPM, LDM, and DPK) were found to be fairly large with more than 30 % of individual tumors with differences exceeding 20 Gy. Differences between MTPM and both 3D-VDM methods were pretty small with 10 and 13 % of the individual tumors with differences higher than 5 Gy, for LDM and DPK methods respectively. The 70% of tumors presented differences between LDM and DPK methods less than 1 Gy.

${\Delta  D}_{mean}^{T_i}$ between both multicompartimental methods (PM-MTPM) and between PM and both 3D-VDM (PM-LDM and PM-DPK) were correlated to differences between TNR and TNR_i_ (rho: 0.96, *p* value: 0.00 for PM-MTPM; rho = 0.97, *p* value = 0.00 for PM-LDM; and rho = 0.97, *p* value: 0.00 for PM-DPK).

**Fig. 8:**
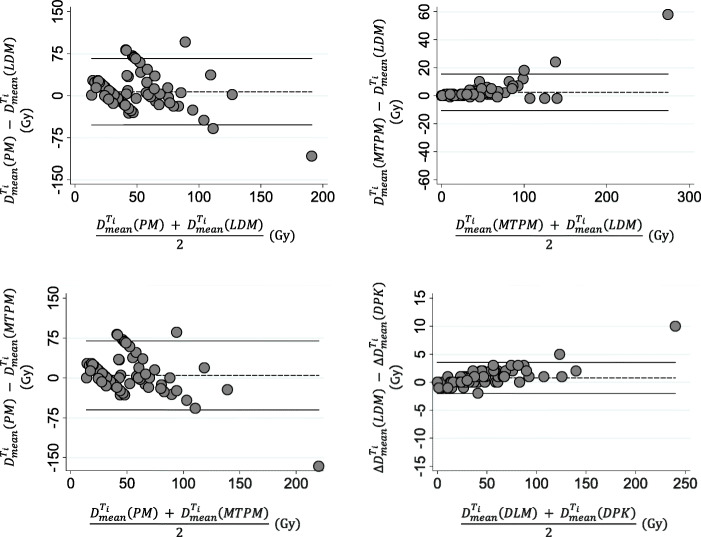
Bland-Altman diagrams representing the relative dose difference in Gy between the studied dosimetry methods versus the mean dose values in Gy for each individual tumor by pairs. The dashed line represents the average of the differences between the studied dosimetry methods, and the black line is the mean differences of ± 2 SD

To assess the agreement between PM and MTPM, PM and LDM, MTPM and LDM and also between both 3D-VDM, in terms of D_mean_, Bland-Altman plots are shown in Fig. 8 for T_i_ and in additional file 3 for TL. Comparisons between both multicompartimental methods (PM and MTPM) and DPK method are presented for Ti in additional file 4. Pearson’s correlation and Lin concordance coefficients for each comparison between dosimetry methods is summarized in Table 5, for both TL and T_i_. The maximum ${\Delta  \mathrm{D}}_{\mathrm{mean}}^{{\mathrm{T}}_{\mathrm{i}}}$ among the studied methods were − 166.0 Gy for PM-MTPM, − 97.8 Gy for PM-DPK, − 107.6 Gy for PM-LDM, 68.2 Gy for MTPM-DPK, 58.4 Gy for MTPM-LDM, and 9.8 Gy for LDM-DPK. $\Delta  {\mathrm{D}}_{\mathrm{mean}}^{{\mathrm{T}}_{\mathrm{i}}}$were higher between PM and the other methods than between MTPM and both 3D-VDM. The lowest differences were found between both 3D dosimetry methods. Significant differences were observed among the all dosimetry methods in terms of ${\mathrm{D}}_{\mathrm{mean}}^{{\mathrm{T}}_{\mathrm{i}}}$ except for PM-MTPM (*p* value = 0.32) and PM-DPK (*p* value = 0.06).

**Table 5:** Bland Altman analysis, Pearson’s correlation, and Lin concordance coefficients, of mean absorbed doses delivered to both tumoral liver and individual tumors volumes among the studied dosimetry methods (PM, MTPM, LDM, and DPK)

	Bland-Altman		Correlation and concordance coefficients	
	$\Delta {D}_{Mean}^{VOI}(Gy)$	Bias [95%CI] (Gy)	*ρ*	*ρ*_c_
*Tumor volume*				
PM-MTPM	− 0.7	− 32.5; 31.0	0.94	0.92
PM-DPK	5.6	− 17.2; 28.5	0.95	0.94
PM-LDM	6.7	− 15.8; 29.2	0.95	0.93
MTPM-DPK	6.3	− 12.7; 25.4	1.00	0.96
MTPM-LDM	7.4	− 14.1; 28.9	0.99	0.94
LDM-DPK	1.1	− 2.0; 4.1	1.00	1.00
*Individual tumors*				
PM-MTPM	4.7	− 60.1; 69.5	0.55	0.55
PM-DPK	7.1	− 52.2; 66.4	0.53	0.50
PM-LDM	7.9	− 50.1; 65.8	0.53	0.50
MTPM-DPK	2.4	− 10.6; 15.4	0.99	0.98
MTPM-LDM	3.2	− 11.8; 18.1	0.99	0.97
LDM-DPK	0.8	− 2.0; 3.5	1.00	1.00

**Fig. 9:**
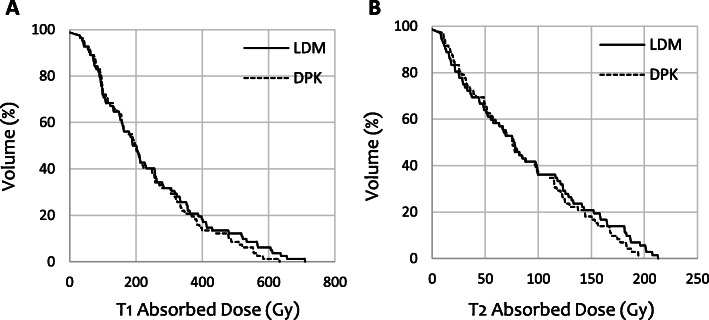
Dose volume histogram curves for each individual tumor, corresponding to T_1_ of patient 9 **a** and T_2_ of patient 10 **b**. LDM dosimetry method is represented by continuous lines and DPK method with the dashed lines

DVH curves calculated by means of LDM and DPK dosimetry methods for two individual tumors are reported in Fig. 9. DVHs correspond to tumors, which yielded the highest differences between both 3D-VDM methods.

The differences between LDM and DPK methods in DVH for each individual tumor volume in terms of D_5_, D_25_, D_50_, D_70_, and D_95_, are reported in Fig. 5. Higher differences were found for D_95_ while D_70_, D_50_, D_25_ and D_5_ showed lower variations in both compartments. The maximum difference was 55.3 Gy, corresponding to D_95_, within T1 of the patient 9 and it is due to a high absorbed dose gradient. The statistical analysis showed that DVH differences between both 3D-VDM methods for T_i_ were statistically significant except for D_25_ (*p* value 0.10).

The mean (range) difference in V_100 Gy_ (%) due to applied LDM or DPK methods was 0.0 (− 1.4, 11.9) %. Significant differences were observed (*p* value < 0.01).

The results of the Bland-Altman analysis to assess differences in DVH in terms of D_5_, D_25_, D_50_, D_70_ and D_95_ are summarized in Table 3, for individual tumor volumes. Pearson’s correlation and Lin concordance coefficients were also reported.

**Fig. 10:**
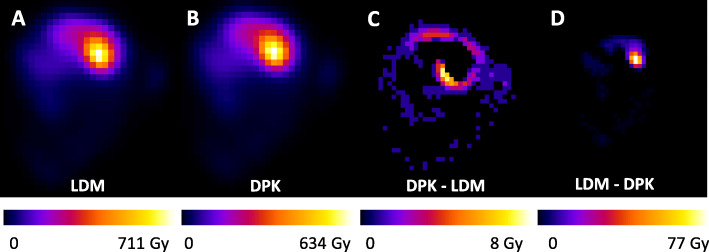
Central coronal slice of the mean absorbed dose distribution map, for patient 9 determined by means of voxel dosimetry methods: LDM **a** and DPK **b**. Voxel based subtraction of parametric images (in Gy): DPK-LDM **c** and LDM-DPK **d**.

Parametric images in terms of absorbed dose for both 3D-VDM methods, and the differences between them by voxel based subtraction are represented in Fig. 10. The images correspond to a patient who yielded the highest differences between both 3D-VDM methods. Figure 10c shows the voxels where parametric image calculated by applying DPK method take values above parametric image determined by means of LDM method, and Fig. 10d presents the voxels where LDM image take values above DPK image. NMSE between absorbed dose maps obtained with each 3D-VDM method was 0.24%, ranged from 0.12 to 0.78 %.

## Discussion

The goal of this study was to evaluate the impact of the dosimetry approach on the resulting ^90^Y-RE pre-treatment absorbed dose based on ^99m^Tc-MAA images. Several available dosimetry methods were compared on a group of 14 patients overall a total of 101 individual tumors. Two different multi-compartmental dosimetry methods (PM and new MTPM) and two 3D voxel based dosimetry methods (DPK and LDM) were studied. Mean absorbed dose, as well as DVH curves, were compared.

For optimal RE treatment, it is essential to be able to predict the absorbed dose to the tumor, healthy liver and lungs. An accurate prediction of these values can help to optimize patient selection and to truly individualize a safe and effective treatment planning [9]. For that, an optimal and reproducible dosimetry approach is not only important but essential. Currently, several dosimetry methods are available and there is no consensus on the best methodology to calculate the absorbed doses [1, 23].

In clinical practice, PM absorbed doses are calculated for the aggregated tumor compartment, and not for each individual tumor. The limitation of PM is the lack of spatial dose information, since it is assumed a homogeneous uptake of ^99m^Tc-MAA in all individual tumors. The new approach of the MTPM method, developed in this study, takes into account the heterogeneity among the individual tumors but no the non-uniform distribution within a given tumor. Voxel-based dosimetry considers voxel-by-voxel heterogeneities between tumors and within each tumor.

Differences between dosimetry approaches are a critical issue and have been explored by other research groups. Pasciak et al. analyzed the effectiveness of several patient-specific dosimetry techniques by means of ^99m^Tc-MAA simulation images of phantoms, such as Monte Carlo, local energy deposition in patient specific phantoms and kernel transport techniques in water [24]. To the best of our knowledge, this is the first study in which a multicompartmental method is implemented for individual tumors. Besides, it is the first time that a comparison among several multicompartimental (PM and MTPM) and 3D voxel dosimetry (LDM and DPK) methods in terms of absorbed doses within each individual tumor is carried out. Other groups have investigated differences on post-treatment PET and Bremsstrahlung images. Thus, Kao et al. studied a technical comparison of partition model and body surface area method with an emphasis on its clinical implications and discuss about future dosimetry techniques for ^90^Y-RE [2]. Mikel et al. in a study published in 2016 quantified differences that exist between glass microsphere package insert standard model (assuming tumor and healthy liver as a single compartment), partition model and Monte Carlo [25]. This group, in a different publication, also assessed differences between four different voxel-based dosimetry methods (Monte Carlo, soft tissue kernel with density correction, soft-tissue kernel and local deposition) for tumor, liver and lung absorbed doses based on ^90^Y-Bremsstrahlung SPECT-CT [26]. Pasciak et al. compared DPK convolution with LDM methods on ^90^Y-PET-CT images using a NEMA Phantom [27].

### Absorbed dose by normal liver

Despite statistical differences were found in mean absorbed dose delivered to the normal liver between all methods, these differences are clinically acceptable. In general, the observed differences were higher between PM and both 3D methods than between LDM and DPK methods. The differences between PM and 3D dosimetry methods were more important within target normal liver than within whole normal liver.

According with Pearson’s correlation and Lin’s concordance coefficients, mean absorbed dose in the healthy liver, calculated by all the dosimetry methods included in this study, were highly correlated and concordant. Besides, only in 1, over the 14 studied patients, the difference is beyond the ± 2 standard deviations lines in the Bland-Altman plots. This concordance, added to the fact that the absolute differences in D_mean_ have no clinical impact, implies that PM and both 3D voxel dosimetry approaches may be used interchangeably for healthy tissue calculations.

The resulting DVH in healthy liver from both 3D-VDM are greatly similar. The extreme difference was found for D_95_ corresponding to patients who have received the higher D_mean_. A high correlation and concordance were found between them and the resulting differences in the absorbed dose have no clinical impact. According to these results, LDM and DPK methods may be indistinctly used for healthy liver absorbed dose prediction.

### Absorbed dose to tumoral liver

In general, the D_mean_ differences (in Gy) found among the dosimetry methods are higher in tumor volumes (TL and T_i_) than in the non-tumoral ones (NL_t_ and NL_w_), which is explained by the greater heterogeneity of the microspheres and the higher dose gradient within the tumor tissue.

For patients with more than a single tumor, a large heterogeneity in the ^99m^Tc-MAA uptake among the individual tumors was found. The COV(TNR_i_) was 56 ± 37 %, ranging from 22 to 113%. This heterogeneity was also observed in the large variability of mean absorbed dose in the tumoral liver compartment determined by MTPM, DPK, and LDM methods, expressed as standard deviation (Table 4).

Despite the fact that no statistical differences in ${D}_{mean}^{T_i}$were found for PM-MTPM and PM-LDM, differences in ${D}_{mean}^{T_i}$ between the PM and the other methods (MTPM, LDM and DPK) were fairly large and may yield a substantial impact in treatment planning. Besides, a poor correlation and concordance between PM and the other studied dosimetry approaches were found. According to these results, this study demonstrates that due to the heterogeneity among tumors, to apply PM method introduces errors in the ${D}_{mean}^{T_i}$ estimation. Therefore, PM method is not the best approach to evaluate de mean absorbed dose in the tumoral parenchyma as this approach neglects the heterogenenity between individual tumors

The strong correlation observed between differences in D_mean_ (PM-MTPM, PM-LDM and PM-DPK) and TNR-TNR_i_ differences suggest that the individual segmentation and evaluation of the individual dose of the tumors plays an essential role in a proper dosimetry estimation.

To introduce the original MTMP method in the clinical practice leads to improve the patient selection; since the patient may not be a good candidate for RE if, due heterogeneous ^90^Y-microspheres distribution, one or more tumors to be treated do not achieve a therapeutic absorbed dose. Moreover, MTMP method could be applied to perform post-treatment dosimetry calculations. For those tumors that have not received a therapeutic absorbed dose, other therapies such as stereotactic body radiation therapy (SBRT), proton therapy, ablation, etc., may be considered.

In specific cases where patients have a single tumor, PM provided acceptable results in D_mean_ within the tumoral parenchyma; however, MTPM or 3D-VDM reduce uncertainty in the absorbed dose calculations for patients with several tumors. Similar conclusion was showed by Mikell et al. [26] who quantified differences between three dosimetry models used for ^90^Y RE: PM, glass microsphere package insert standard model and Monte Carlo.

Differences in ${D}_{mean}^{T_i}$ between MTPM and both 3D-VDM were substantially lower than differences between PM and any 3D-VDM, as MTPM introduces more information regarding the different ^99m^Tc-MAA uptake in each tumor. Although differences between MTPM and both LDM and DPK methods were statistically significant, a strong correlation and concordance were found, and the differences in ${D}_{mean}^{T_i}$ have no impact in clinical management. Consequently, MTPM or 3D dosimetry approaches may be indistinctly used to calculate D_mean_ within individual tumor volumes.

The principal advantage of MTPM over 3D-VDM is its easier implementation, because no algorithm or specific software dosimetry is necessary for dose calculation and therefore it has a greater availability in daily clinical practice. Despite applying MTPM is laborious in those cases in which the number of individual tumors is large, the same contours must be delineated to perform 3D voxel dosimetry. Taking this into account, in centers where it is not possible to perform a dosimetry based on 3D voxel methods, to implement MTPM is recommended in order to improve the accuracy of D_mean_ calculation.

The differences between LDM and DPK methods in${D}_{mean}^{T_i}$ and DVH are statistically significant. However, taking into account the uncertainty associated with the dose calculation procedure, these differences are clinically acceptable and have no impact in treatment planning. An almost perfect correlation and concordance were found. Then either 3D dosimetry approaches may be indistinctly used to calculate D_mean_ within individual tumor volumes.

Although global differences in terms of absorbed dose in Gy and NMSE are small, important voxel-by-voxel differences have been observed (maximum difference: 77 Gy). This is a consequence of the high absorbed dose gradient. Since DPK method takes into account the energy transport to adjacent voxels, it was expected that DPK tended to provide higher absorbed doses than LDM in outer edge of the uptake region, while LDM tended to provide higher absorbed doses in the central area, as the results of this study show (Fig. 10).

The need to compare dosimetry performed on ^99m^Tc-MAA SPECT-CT pre-therapy images to post-therapy images, in order to study the effectiveness of ^99m^Tc-MAA, is pointed out by many studies [28–31]. As this study suggest, in some situations, the dosimetry approach has a substantial impact on the resulting absorbed doses; therefore, considering the differences in absorbed doses due to different dosimetry methods is essential not only to compare pre and post dosimetry calculations but also to interpret different clinical studies.

As it is shown in this study, the differences in ${D}_{mean}^{N{L}_w}$between PM and both 3D-VDM that we found in Bland-Altman analysis (− 0.3 and 1.7 Gy for DPK; − 0.1 and 2.4 Gy for LDM) are lower than the differences between pre and post-dosimetry comparison (− 7.4 and 9.1 Gy) reported by Richetta et al. [29]. However, the differences in ${D}_{mean}^{T_i}$between PM and the other dosimetry approaches that we found in Bland-Altman analysis (− 60.1 and 69.5 Gy for MTPM, − 52.2 and 66.4 Gy for DPK; − 50.1 and 65.8 Gy for LDM) are comparable to the differences between pre and post-dosimetry comparison (− 79 and 68 Gy) reported by Richetta et al. [29].

Therefore, based on the results of this study, to use the same approach in predictive and post-treatment dosimetry calculation is recommended, in order to be able to make a comparison between them in terms of D_mean_ within both tumoral and non-tumoral parenchyma. Furthermore, to take into account the differences in D_mean_ across dosimetry methods is essential in interpreting clinical studies that use different dosimetry approaches.

### Limitations and future perspectives

There are other methodological variables, not included in this work, which may be source of differences in the absorbed dose results. Image registration and VOIs segmentation may be a limiting factor for all dosimetry methods, due to large absorbed dose gradients presents in RE treatments, especially important near liver-lung interface [26]. The calibration factor used to convert counts to Bq/cm^2^ may be determined not only applying a self-calibration factor, as described in this work, but also by means of the evaluation of SPECT system sensitivity [32]. Voxel-based dosimetry may suffer from bias related to acquisition and reconstruction parameters, partial volume effect, etc. [24].

## Conclusion

This work shows that significant differences exists among the studied pre-treatment dosimetry approaches (PM, MTPM, LDM, and DPK methods) for ^90^Y RE treatments. However, these differences do not yield a substantial impact in treatment planning for healthy tissue from a clinical point of view and different dosimetry approaches may be applied indistinctly.

Due to the large heterogeneity found among individual tumors, an individual segmentation and evaluation of the tumors plays an essential role in a proper dosimetry estimation. Therefore, in patients with multiple tumors, to apply PM method is not recommended in tumoral parenchyma since the mean absorbed dose are estimated within the aggregated tumor compartment, and the 3D dosimetry methods or the new MTPM should be applied.

Moreover, when a 3D voxel based dosimetry method is not available, MTPM is the best option to estimate the mean absorbed dose within each tumor. Both LDM and DPK methods may be indistinctly used.

Although this study showed that some methods are interchangeably, to make an optimal comparison between absorbed dose values from different publications or even from the same study, i.e., pre and post-dosimetry, is essential to take into account differences in the absorbed doses caused by the adoption of a different dosimetry approach.

## Supplementary Information

**Additional file 1:.**

**Additional file 2:.**

**Additional file 3:.**

**Additional file 4:.**

## Data Availability

The datasets used and/or analyzed during the current study are available from the corresponding author on reasonable request.

## Abbreviations

3D: Tridimensional
3D-VDM: 3D-voxel dosimetry methods
($\Delta {D}_{Mean}^{VOI}$): Mean absorbed dose difference within a VOI
ρ: Pearson correlation coefficient
ρ_c_: Lin concordance coefficient
A(^99m^Tc): ^99m^Tc Administered Activity
A(^90^Y): ^90^Y Administered Activity
A_voxel_(^90^Y): ^90^Y Activity within a voxel
${\overset{\sim }{A}}_{voxel}\left({}^{90}Y\right)$: ^9o^Y Cumulative Activity within a voxel
${\overset{\sim }{A}}_{voxe{l}_s}\left({}^{90}Y\right)$: ^90^Y Cumulative Activity within a source voxel
C_lung_: Lung total counts
${C}_{N{L}_t}$: Target normal liver total counts
C_Ti_: Individual tumor total counts
C_TL_: Tumoral liver total counts
C_voxel_(^99m^Tc): Total counts within a voxel generated by ^99m^Tc
C_WL_: Whole liver total counts
C_WL_(^99m^Tc): Whole liver total counts generated by ^99m^Tc
COV: Coefficient of variation
CT: Computed tomography
D_5_: Minimum absorbed dose to 5% in the corresponding VOI
D_25_: Minimum absorbed dose to 25% in the corresponding VOI
D_50_: Minimum absorbed dose to 50% in the corresponding VOI
D_70_: Minimum absorbed dose to 70% in the corresponding VOI
D_95_: Minimum absorbed dose to 95% in the corresponding VOI
D_mean_: Mean absorbed dose
${D}_{Mean}^{T_i}$: Individual tumor mean absorbed dDose
${D}_{Mean}^{TL}$: Tumoral liver mean absorbed dose
${D}_{Mean}^{N{L}_t}$: Target normal liver mean absorbed dose
${D}_{Mean}^{N{L}_w}$: Whole normal liver mean absorbed dose
${D}_{voxe{l}_t}\left({}^{90}Y\right)$: ^90^Y Mean absorbed dose within the target voxel
DICOM-RT: Digital Imaging and Communications in Medicine - Radiation Therapy
DPK: Dose Point Kernel
DVH: Dose volume histogram
〈*E*_*β*_(^90^*Y*)〉: Deposited β-energy per disintegration in average
FWHM: Full width half maximum
LDM: Local deposition method
LEHR: Low energy high resolution
LSF: Lung shunt fraction
*M*_*voxel*_: Voxel mass
MAA: Macroaggregated albumin
MIRD: Medical internal radiation dose
MRI: Magnetic resonance imaging
MTPM: Multi-tumor partition model
n: Number or tumors
NMSE: Normalized mean square error
NL_t_: Target normal liver
NL_w_: Whole normal liver
p_i_: Ith voxel of the LDM parametric image
PM: Partition model
PTV: Planning target volume
RE: Radioembolization
*S*(*voxel*_*t*_ ← *voxel*_*s*_): Absorbed dose within the target voxel per unit of cumulative activity within the source voxel
SPECT: Single photon emission computed tomography
T_1/2_(^90^*Y*): Physical ^90^Y half life
T_i_: Individual tumors
TL: Tumoral liver
TNR: Tumor to non-tumor ratio
TNR_i_: Individual tumor to non-tumor ratio
V_20_: Percentage of corresponding VOI receiving at least 20 Gy
V_100_: Percentage of the tumor volume receiving at least 100 Gy
${V}_{N{L}_t}$: Target normal liver volume
${V}_{N{L}_w}$: Whole normal liver volume
V_Ti_: Individual tumor volume
V_TL_: Tumoral liver volume
VOI: Volume of interest
WL: Whole liver
x_i_: Ith voxel of the DPK parametric image
