# Supplementary figures and images for: Impact of the dosimetry approach on the resulting 90Y radioembolization planned absorbed doses based on 99mTc-MAA SPECT-CT: is there agreement between dosimetry methods?
Source: EJNMMI Phys. 2020 Dec 7;7:72. doi: 10.1186/s40658-020-00343-6 (PMC7721939; doi:10.1186/s40658-020-00343-6)

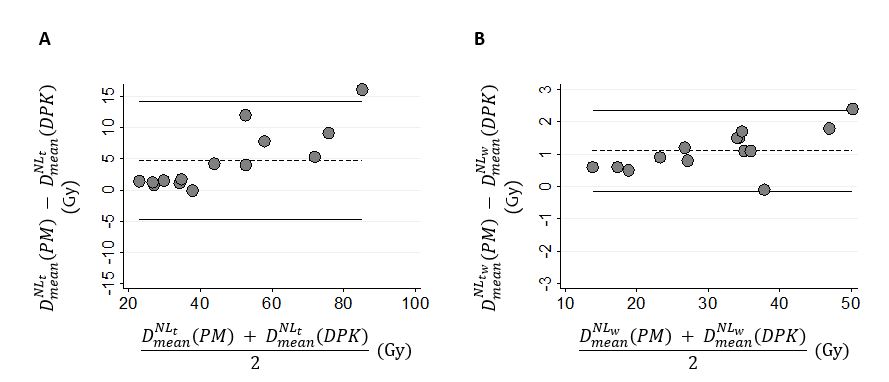

Supplement: Supplementary file 2 — Additional file 2:. [file 40658_2020_343_MOESM2_ESM.jpg]

## Slide 1
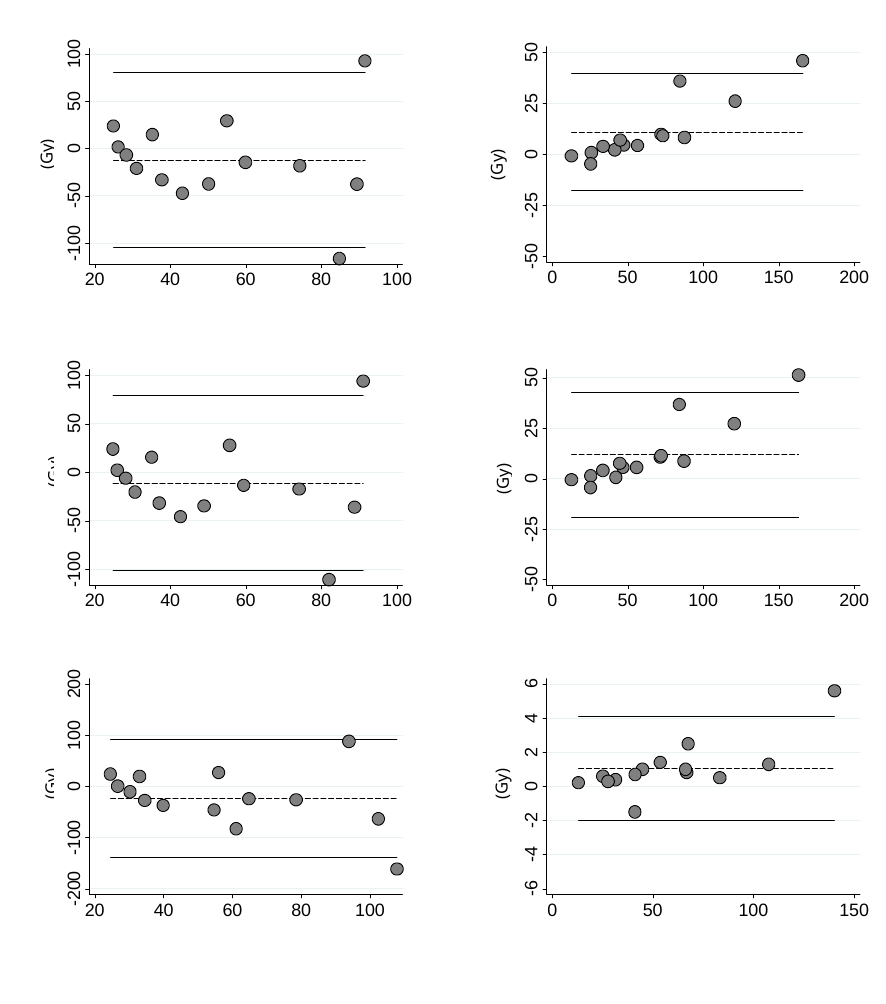

Supplement: Supplementary file 3 — Additional file 3:. [file 40658_2020_343_MOESM3_ESM.pptx]

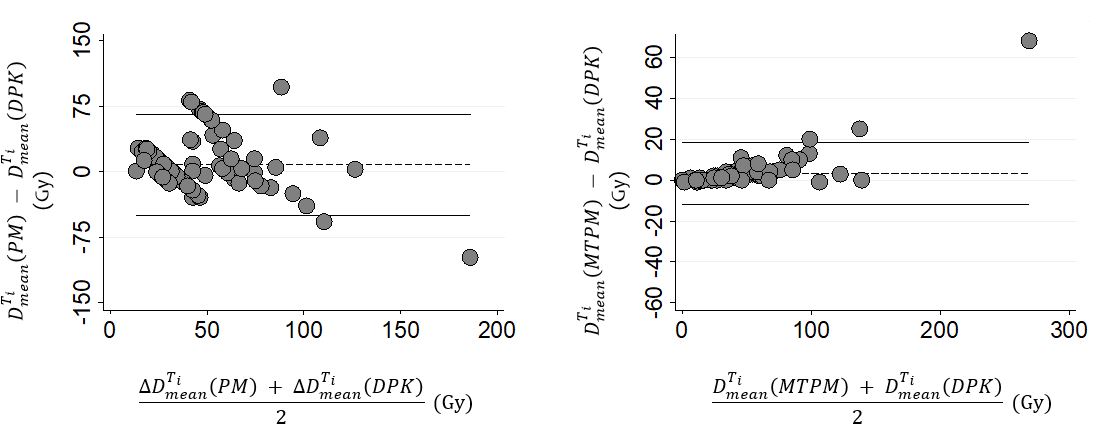

Supplement: Supplementary file 4 — Additional file 4:. [file 40658_2020_343_MOESM4_ESM.jpg]
